# Modulating mechanisms of adverse childhood experiences in a mindfulness-based intervention: preliminary insights from an opioid use disorder study

**DOI:** 10.3389/fpsyg.2025.1529106

**Published:** 2025-04-30

**Authors:** Diane Joss, Joseph Rosansky, Paula Gardiner, Robert R. Edwards, Roger D. Weiss, Vitaly Napadow, Zev Schuman-Olivier

**Affiliations:** ^1^Center for Mindfulness and Compassion, Cambridge Health Alliance, Malden, MA, United States; ^2^Department of Psychiatry, Harvard Medical School, Boston, MA, United States; ^3^Pain Management Center, Department of Anesthesiology, Brigham and Women’s Hospital, Boston, MA, United States; ^4^Division of Alcohol, Drugs, and Addiction, McLean Hospital, Belmont, MA, United States; ^5^Schoen and Adams Discovery Center for Chronic Pain Recovery, Department of Physical Medicine and Rehabilitation, Spaulding Rehabilitation Hospital, Boston, MA, United States

**Keywords:** meditation, addiction, depression, anxiety, trauma, stress

## Abstract

**Introduction:**

Adverse childhood experiences (ACE) are transdiagnostic developmental risk factors for various mental and physical health issues, including Opioid Use Disorder (OUD). Existing research demonstrated ACE not only affects the onset, severity, and comorbidity of disorders, but also affects treatment responses. To investigate whether and how ACE modulates treatment effects of Mindfulness Based Intervention (MBI), we conducted secondary analysis on the longitudinal data from a recent clinical trial on the effects of a MBI during outpatient buprenorphine treatment.

**Methods:**

Using data from a RCT that randomized (1:1) a total of 196 patients with OUD into a live online group intervention with either a 24-week MBI or a matched recovery support control group, we conducted temporal path analysis with the following outcome measures: self-critical rumination, pain catastrophizing, pain interference, severity of depression and anxiety.

**Results:**

Both treatment arms had significant reduction of all symptom measures, but the MBI arm had a significant mechanistic path of ACE (baseline) ➔Self-Critical Rumination (week 8)➔Pain Catastrophizing (week 16) ➔ Pain Interference (week 24), which was not significant in the control arm. Only in the MBI arm, ACE severity was significantly correlated with score reductions of Self-Critical Rumination (week 8), which was not significant in the control arm.

**Conclusion:**

ACE modulated treatment responses to MBI, through a mechanistic path in which symptom changes of Self-Critical Rumination was a mediator between ACE and psychological symptom changes of pain catastrophizing and pain interference, suggesting Self-Critical Rumination can be considered as a therapeutic target in future intervention development.

## 1 Introduction

Adverse Childhood Experiences (ACE) have been shown to be a major risk factor for developing various psychological and medical symptoms throughout the lifespan (Felitti et al., 1998), with earlier onset (Sonu et al., 2019), more comorbidity (Lovis-Schmidt et al., 2024), and less responsivity to traditional pharmacological or behavioral interventions (Nanni et al., 2012; Nemeroff et al., 2003). Opioid Use Disorder (OUD), as an example of comorbid psychological and somatic symptoms, has been a devastating public health crisis (Volkow and Blanco, 2021). Patients with high ACE tend to have earlier onset (Swedo et al., 2020) and higher symptom severity of OUD (Stein et al., 2017). “Pain interference,” a psychological measure of pain-related psychosocial and functional impairments, has been shown to be a risk factor for developing OUD (Blanco et al., 2016), and does not usually improve during buprenorphine treatment alone (Edwards et al., 2022). Therefore, improving mechanistic understanding on the relationships between ACE and pain interference, and identifying potential psychological therapeutic targets, are key steps in developing effective interventions (Yamin et al., 2024) for OUD and chronic pain treatment for the population with high ACE.

Emerging research over the past decade suggests that individuals with high ACE might particularly benefit from Mindfulness-Based Interventions (MBIs). A large clinical trial (*n* = 274) on relapse prevention for recurrent depression demonstrated Mindfulness Based Cognitive Therapy (MBCT) was more effective than control conditions for patients with above median level childhood trauma (Williams et al., 2014). Our recent pilot studies with trauma-sensitive MBI demonstrated improved perceived stress and anxiety among young adults with ACE (Joss et al., 2019, 2021), and that ACE levels mediated and moderated the brain-behavior associations between adaptive neural changes and improvement in psychological symptoms (Joss et al., 2021, 2024a,b). Therefore, we hypothesize that ACE levels could modulate treatment responses to MBIs.

Our recent research indicates that MBIs might be particularly beneficial for patients with high ACE levels in terms of reducing “*Self-Critical Rumination”* (SCR) (Joss et al., 2025). SCR refers to maladaptive repetitive thinking of past failures and inadequacies without consideration for improvement or problem-solving (Kolubinski et al., 2021). Developmentally, the psychological trait of SCR often arises among people with high levels of ACE (Kannan and Levitt, 2013; Lassri et al., 2018; Shahar et al., 2015), due to internalized criticism from others, proneness to shame and guilt (Shahar et al., 2015) or coping with self-blame (Tanzer et al., 2021). Because the psychological experience of chronic pain is often accompanied by rumination (Edwards et al., 2011) and shame (Coady et al., 2024), trait SCR is likely a risk factor for developing symptoms such as pain catastrophizing (Quartana et al., 2009), depression (Surah et al., 2014), anxiety (Kopala-Sibley et al., 2015) and pain interference (Amtmann et al., 2010) in response to pain experiences. Therefore, we hypothesize that SCR is a mediator between ACE and pain-related psychological symptoms and can be one of the first psychological elements to respond to MBIs.

“Pain catastrophizing” is another psychological symptom that can also influence pain interference. The concept “pain catastrophizing” captures maladaptive psychological responses to anticipated or actual pain, such as pain-related rumination, magnification of pain intensity, and sense of helplessness with pain conditions (Quartana et al., 2009). Pain catastrophizing has been shown to be a significant predictor for patients to experience pain related psychosocial functional impairments because of the psychological distress, negative beliefs, and maladaptive thought patterns (Hanley et al., 2008; Jang et al., 2018). Because SCR reflects negative beliefs (Mills et al., 2007) and ruminative thought patterns (Sethi, 2024), it’s likely a precursor for developing pain catastrophizing (Quartana et al., 2009). Therefore, we hypothesized that SCR and pain catastrophizing were sequential mediators between ACE and pain interference.

To test this hypothesis, we conducted a secondary analysis with the longitudinal data from a recent clinical trial (Rosansky et al., 2024) in which pain interference was an *a priori* secondary outcome, designed to compare the effects of a 24-week live online trauma-informed Mindful Recovery OUD Care Continuum (M-ROCC) program vs. a live-online recovery support group as an evidence-based active control during outpatient buprenorphine treatment for OUD (Rosansky et al., 2024). This manuscript reports the treatment effects on pain interference and investigates the mechanistic relationship of SCR and pain catastrophizing as sequential mediators between ACE and treatment effects on pain interference.

## 2 Methods

### 2.1 Subject enrollment, intervention, and assessments

This study is registered on ClinicalTrials.gov (NCT04278586) and approved by the Cambridge Health Alliance (CHA) Institutional Review Board (IRB). Participants were recruited through social media advertisements, community healthcare centers, as well as online telemedicine providers (Bosse et al., 2022; Rollston et al., 2023). Inclusion criteria were between 18 and 70 years old, having been prescribed a stable dose of buprenorphine for at least 4 weeks, diagnosed with OUD, and either less than 90 days abstinent (from opioids, benzodiazepines, cocaine, methamphetamine, or alcohol) OR having comorbid anxiety. Exclusion criteria included active psychosis, bipolar I disorder, suicidality/self-injurious behavior, cognitive impairment, current participation in another research study, past mindfulness group experience, expected inpatient treatment or incarceration, or inability to participate in group sessions.

Subjects were randomized to the M-ROCC or the control arm in a 1:1 ratio. All group meetings of both intervention programs were time-and attention-matched and conducted in separate virtual Zoom spaces. Each group meeting session was ~90 min. The M-ROCC intervention included cultivating mindfulness of the body, breathing, thoughts, and emotions, as well as skills for mindful behavior change, interpersonal mindfulness practice, self-compassion and emotion regulation, as well as developing OUD recovery skills such as mindful savoring and urge surfing (Rosansky et al., 2024). The active control intervention was based on best practices in group-based opioid treatment (GBOT) (Sokol et al., 2019; Sokol et al., 2018b), designed to offer evidence-based techniques while fostering a sense of accountability, shared identity and supportive community (Sokol et al., 2018a), with a curriculum using evidence-based therapeutic elements including cognitive-behavioral therapy (Moore et al., 2016), motivational interviewing (Smedslund et al., 2011), community reinforcement (Brigham et al., 2014; Meyers et al., 2011), and twelve-step facilitation (Nowinski et al., 1995). For treatment fidelity, all online group sessions were audio-recorded with at least 10% recordings randomly audited by trained supervisory personnel with timely feedback (Crane et al., 2013).

### 2.2 Measures

All subjects filled out a battery of psychological questionnaires through a secure online platform REDCap (Harris et al., 2009) at pre-intervention baseline, as well as 8 weeks, 16 weeks and 24 weeks during the 24 week intervention; the following questionnaires were analyzed in this secondary analysis study:

The ACE questionnaire (Felitti et al., 1998) was used to measure the severity of ACE, which includes 10 items describing scenarios of childhood abuse, neglect, and household dysfunction during the first 18 years of life, with a score range of 0–10. Prior research has reported Cronbach alpha of 0.70 (Oláh et al., 2023).

The Patient-Reported Outcomes Measurement Information System (PROMIS®) Adult Short Form v1.0 with Pain Interference 8a (PROM-PI) (Amtmann et al., 2010), anxiety (PROM-A) and depression (PROM-D) modules (Pilkonis et al., 2014). The pain interference module has 8 items on the extent to which pain hinders engagement with social, cognitive, emotional, physical, and recreational activities in the past 7 days, it’s a highly reliable instrument with Cronbach alpha of 0.99 (Amtmann et al., 2010). The anxiety and depression modules each include 8 items on anxiety or depression symptoms in the past 7 days, with Cronbach alpha of 0.97 (Schalet et al., 2014) and 0.92 (Pilkonis et al., 2014) respectively.

Self-Critical Rumination Scale (SCRS) (Smart et al., 2016) is a 10-item self-report questionnaire responded on a 4-point Likert scale to indicate “how well each item describes you,” with example items include: “I always seem to be rehashing in my mind stupid things that I’ve said or done.” “I cannot stop thinking about how I should have acted differently in certain situations.” SCRS has high internal consistency with alpha = 0.92 (Smart et al., 2016).

Pain Catastrophizing Scale (PCS) (Sullivan et al., 1995) is a 13-iterm self-report questionnaire responded on a 5-point Likert scale. PCS has three subscales: rumination, magnification and helplessness, with alpha = 0.87 for total score (Osman et al., 2000).

### 2.3 Statistical analysis

Linear mixed effects model analyses (Lindstrom and Bates, 1988) were conducted with the “*nlme*” and “*MuMIn*” packages in statistical software R. The scores of each questionnaire were used as the dependent variable for each model, with “group” (i.e., MBI vs. control) and “time point” (i.e., baseline (week 0), week 8, week 16 and week 24) as independent variables, and group by time interaction was the effect of interest, with age, sex, and race as covariates; separate variance for each group and time point was used, and “REML” method was chosen to maximize the restricted log-likelihood.

To identify mechanistic paths of symptom changes, we employed path analysis using maximum likelihood estimation with the “sem” function in the *lavaan* package for R Statistics. We hypothesized a cascade of temporal sequence of symptom changes, i.e., ACE scores modulate ΔSCRS, which then affect ΔPROM-D or ΔPROM-A, which then affect ΔPCS which eventually affects ΔPROM-PI. Therefore, we used Δ SCRS at week 8, ΔPROM-D or ΔPROM-A at week 16, and ΔPCS and ΔPROM-PI scores at week 24. We used the “tests of joint significance” principle (Leth-Steensen and Gallitto, 2016) to identify significant indirect effects. Standardized beta weights were calculated for each significant indirect effect and total indirect effect sizes using the “sem” function in *lavaan* (Rosseel, 2012), with model fit parameter thresholds Comparative Fit Index (CFI) ≥ 0.95 and Standardized Root Mean Square Residual (SRMR) ≤ 0.08 (Rosseel, 2012; Schreiber et al., 2006). To facilitate comparisons of the magnitude of effects, we reported absolute values for all standardized beta weights. The Benjamini-Hochberg (Cribbie, 2007) procedure with a false discovery rate (FDR) of alpha = 0.05 was utilized. We employed complete case analysis to handle missing data in the path analysis, with final sample sizes of *n* = 42 and *n* = 54 utilized for the M-ROCC and control group, respectively.

## 3 Results

### 3.1 Demographics and clinical characteristics

A total of 196 eligible consented patients were equally randomized (1:1) into M-ROCC or control group interventions. The two groups did not differ in demographic variables such as gender ratio, age, racial composition, and employment status (Table 1). They also did not differ in ACE levels and baseline scores of SCRS, PCS, PROM-D, PROM-A, and PROM-PI.

**Table 1:** Subject baseline demographic and clinical characteristics at the time of randomization.

Baseline characteristics	MBI (*n* = 98)	Control (*n* = 98)	*p*-value	Total (*n* = 196)
Female, *N* (%)	60 (61.2%)	59 (60.2%)	0.6	119 (60.7%)
Age (years), mean (SD)	42.2 (10.4)	40.9 (10.3)	0.87	41.0 (10.3)
Race, *N* (%)			0.63	
American Indian or Alaska Native	1 (1.0%)	0 (0%)		1 (0.5%)
Asian	0 (0%)	1 (1.0%)		1 (0.5%)
Black, Haitian, or African American	1 (1.0%)	0 (0%)		1 (0.5%)
Native Hawaiian, or Other Pacific Islander	1 (1.0%)	0 (0%)		1 (0.5%)
White	90 (91.8%)	90 (91.8%)		180 (91.8%)
More than one race	2 (2.0%)	3 (3.1%)		5 (2.5%)
Unknown or not reported	3 (3.1%)	4 (4.1%)		7 (3.6%)
Ethnicity, *N* (%)			0.36	
Hispanic, Spanish, or Latinx origin	10 (10.2%)	6 (6.1%)		16 (8.2%)
Not Hispanic, Spanish or Latinx	88 (89.8%)	91 (92.9%)		179 (91.3%)
Unknown or not reported	0 (0%)	1 (1.0%)		1 (0.5%)
Unemployment Status, *N* (%)	18 (18.4%)	17 (17.4%)	0.85	35 (17.9%)
Total ACEs, mean (SD)	4.9 (2.8)	4.8 (2.8)	0.76	4.9 (2.8)

### 3.2 Treatment effects on outcome measures

There was no significant effect of group, or group by time interaction effect with any of the outcome measures. There was significant effect of time with all outcome measures (*p* < 0.001). At the end of program week 24, both treatment arms had significant reductions with PROM-PI (MBI: *d* = −0.35, control *d* = −0.39, *p* < 0.05, FDR corrected) without significant difference.

### 3.3 ACE scores modulate treatment responses to MBI only

**Figure 1:**
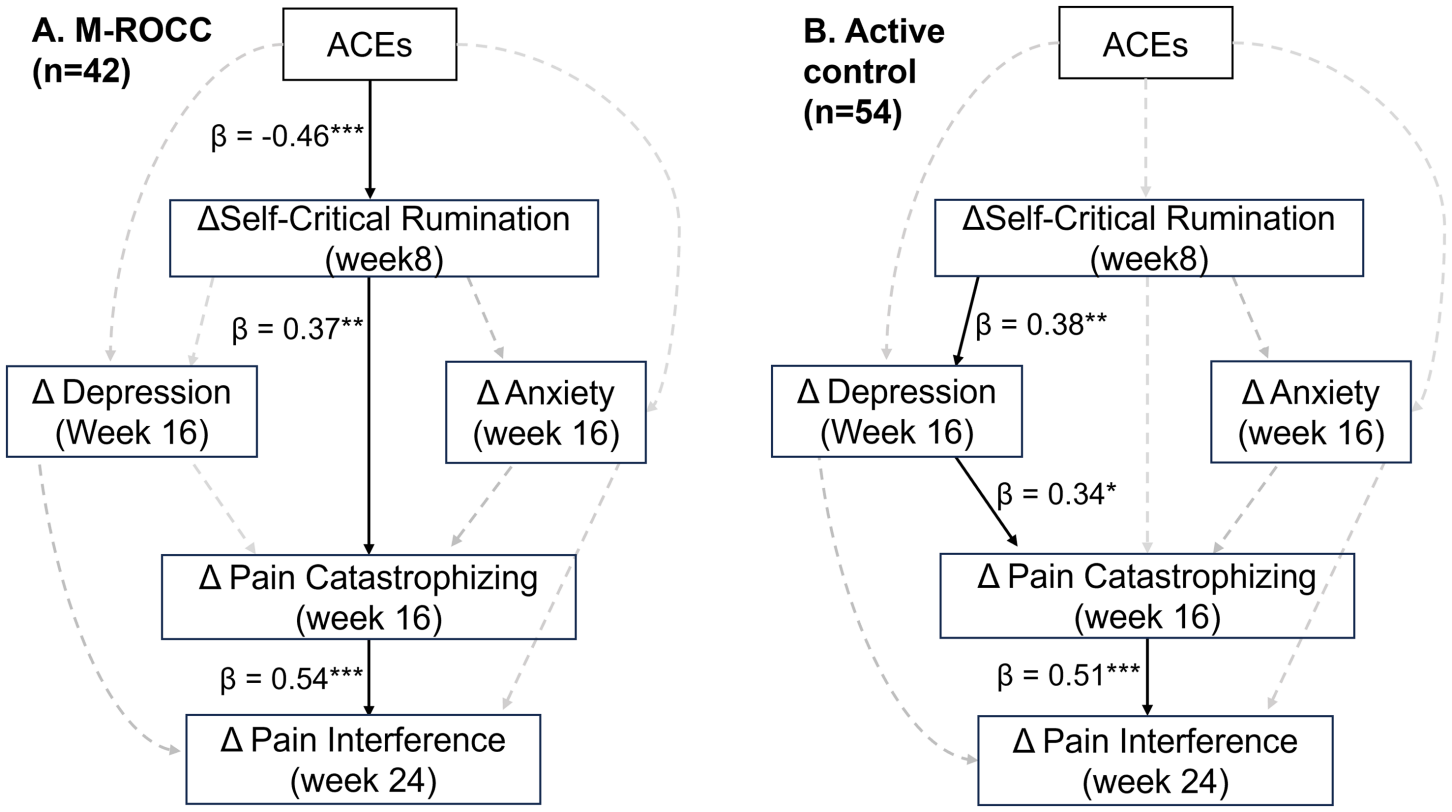
Different temporal paths of symptom changes in the **(A)** M-ROCC and **(B)** active control group.

Path analyses revealed that despite lack of group difference on symptom changes, the mechanistic pathways are different between the two groups. The path models for both groups had good model fit. The path model of M-ROCC group had CFI = 0.95 and SRMR = 0.06, whereas the model of the control group had CFI = 0.99 and SRMR = 0.04. The M-ROCC group had a significant pathway of ACE➔ΔSCRS (week 8) ➔ΔPCS (week 16) ➔ΔPROM-PI (week 24), which was not significant in the control group. Only in the M-ROCC group, ACE had a direct effect on ΔSCRS (week 8) (*β* = −0.46, *p* < 0.001), which was not significant in the control group. Also, only in the M-ROCC group, ΔSCRS (week 8) had a direct effect on ΔPCS (week 16) (*β* = 0.37, *p* < 0.01) without going through ΔPROM-D or ΔPROM-A. In comparison, the control group only had a path of ΔSCRS (week 8) ➔ΔPROM-D (week 16) ➔ΔPROM-PI (week 24) (*p* < 0.05) (see Figure 1).

**Figure 2:**
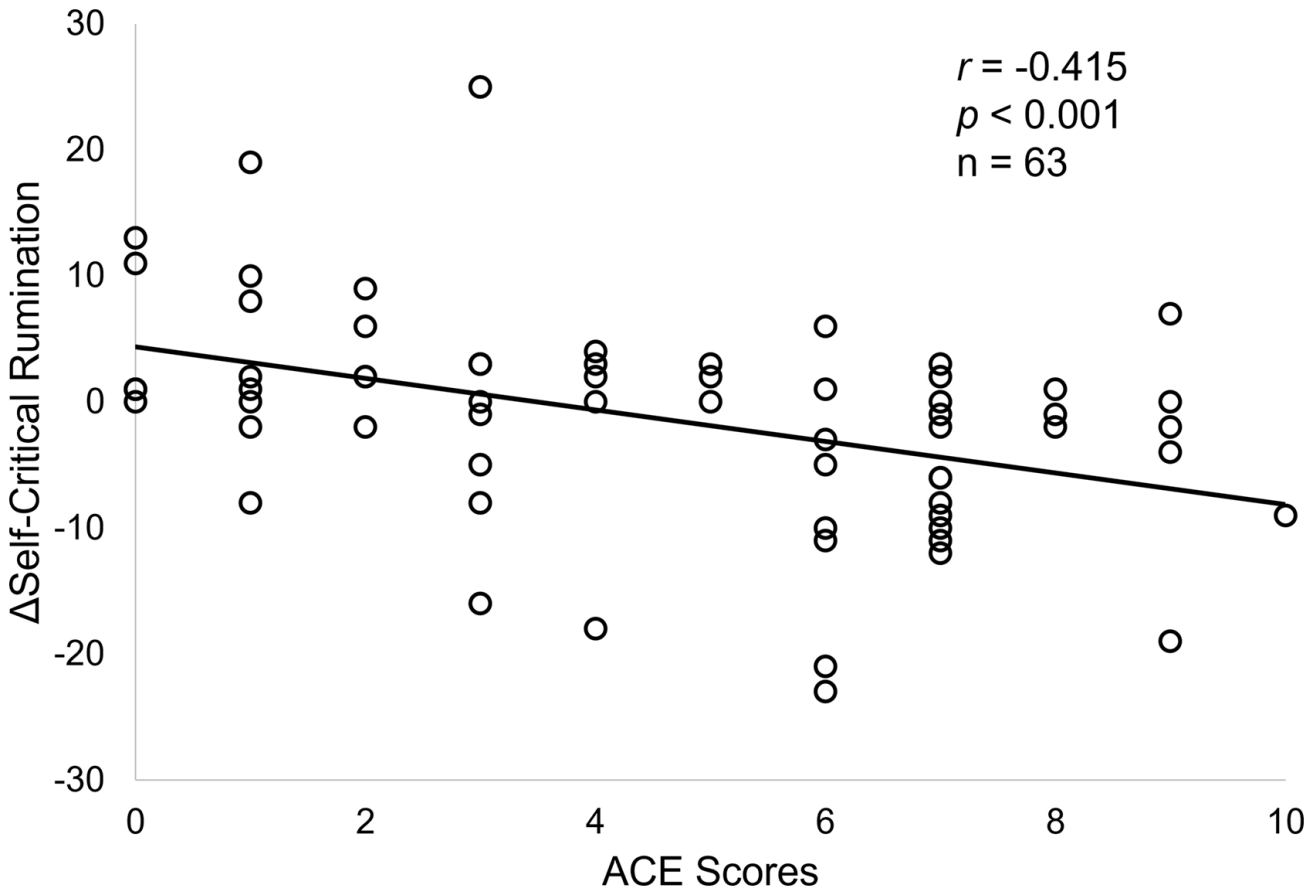
Correlations between ACE scores and ΔSCRS at week 8 in the M-ROCC treatment arm.

*Post hoc* Pearson correlation analyses demonstrated significant correlations between ACE and ΔSCRS (week 8) only within the M-ROCC arm (*r* = −0.415, *p* < 0.001, Figure 2), but not the control arm. There was also a significant correlation between ΔSCRS (week 8) and ΔPCS (week 24) within the M-ROCC arm (*r* = 0.419, *p* = 0.004), but not in the control group.

Using a similar approach as in prior research (Joss et al., 2025; Williams et al., 2014), *post hoc* two-sample t-tests with a median split (ACE = 5) demonstrated that, within the M-ROCC arm, patients with ACE ≥5, compared to patients with ACE < 5, had significantly more reduction of ΔSCRS (week 8) (*t* = −3.361, *p* = 0.001), but similar analysis with median split (ACE = 5) within the control arm did not find any significant difference. However, among all patients with ACE ≥5 from both arms, there was no significant difference between M-ROCC and controls in terms of ΔSCRS (week 8). Similar sub-group analysis with the ΔPROM-PI (week 24) measure did not find any significant differences within either arm or between the two arms.

## 4 Discussion

Findings from this longitudinal study demonstrated that (1) ACE only modulated treatment response to MBI, but not the control intervention; (2) ΔSCRS significantly mediated the relationship between ACE and ΔPCS in the MBI arm, but not in the control arm; (3) in the control arm, ΔSCRS also showed effects on ΔPCS and ΔPROM-PI.

The finding of lack of significant group by time interaction effect with any of the outcome measures is consistent with similar findings of the two treatment arms not having significant differences in the primary outcome measure of abstinence from illicit opioid use from the parent clinical trial (Schuman-Olivier et al., 2025), as well as other similar findings in the MBI literature (Goldberg et al., 2022; Goldberg et al., 2018). Accumulating amounts of research suggest that MBIs tend to have small effects sizes or no significant differences compared to active controls, which was repeatedly found from various disease conditions, e.g., addiction, anxiety, depression, and pain (Goldberg et al., 2022; Goldberg et al., 2018). A recent review of 44 meta-analysis of MBI RCTs (*k* = 336 RCTs, total *n* = 30,483 participants) revealed that MBIs only have consistent superiority compared to passive controls, but tend to have small effect sizes that are often statistically indistinguishable from active controls (Goldberg et al., 2022). Therefore, the lack of group differences of post-intervention symptom changes in the current RCT is consistent with prior findings in the MBI literature (Goldberg et al., 2022). However, emerging mechanistic studies have demonstrated that despite having similar effects on outcome measures, MBIs and active controls can have different psychological (Joss et al., 2024c) and neural (Joss et al., 2024a; Joss et al., 2024b) mechanisms. Therapeutic effects of active control conditions are often due to non-specific factors such as therapeutic alliance and peer support (Chatoor and Kurpnick, 2001), which was not quantified in the present study. Nevertheless, these mechanistic findings indicate the therapeutic effects of MBIs might have the potential to have better generalizability and reliability because of the clear mechanisms, which can be further evaluated in large-scale implementation science research (Bauer and Kirchner, 2020), and the knowledge of psychological mechanisms also provides opportunities for further adaptation and improvement of MBIs.

Consistent with prior findings of different psychological mechanisms between MBI and active control for other outcome measures in other patient populations (Joss et al., 2024c; Williams et al., 2014), findings from this study also revealed different mechanisms between MBI and control, in which ACE and ΔSCRS only modulated ΔPCS and ΔPROM-PI in the MBI arm but not in the control arm. In the MBI treatment arm, patients with higher levels of ACE had more reduction of SCR as demonstrated by a significant correlation between the two variables as well as significant differences between the subset of patients with above vs. below median ACE levels, neither of which were significant in the control arm.

There is limited prior research in the MBI literature on how ACE affects treatment effects, and there are still many outstanding questions (Joss and Teicher, 2021). The following two questions are fundamental in theory: (1) do patients with high ACE levels, compared to patients with low ACE levels, benefit more from MBIs? (2) do patients with high ACE levels respond better to MBIs compared to other treatments? The present study provided some tentative answers to the first question with findings from the M-ROCC arm showing that patients with higher ACE levels had more reduction of ΔSCRS (week 8), although this pattern did not hold for the ΔPROM-PI (week 24) outcome measure. This limited finding indicates that MBI may be particularly beneficial for addressing SCR among childhood trauma survivors, an effect that was also supported by findings from a recent single arm study that MBI induced significantly more improvement with self-judgment and self-compassion among the subset of patients with above-median-level of childhood trauma compared to the rest of patients (Joss et al., 2025). The M-ROCC program in the current study also incorporated teaching of mindfulness and self-compassion similar to the self-compassion-focused MBI used in the prior study (Joss et al., 2025), which may explain its similar effect on SCR. However, the M-ROCC program was not designed for treating psychological symptoms of pain, therefore it is still inconclusive whether a more customized MBI program targeting chronic pain might also demonstrate stronger therapeutic effects for the subset of patients with higher ACE levels within the MBI arm.

Answering the second question “do patients with high ACE levels respond better to MBIs compared to other treatment options?” would require a much larger specifically designed RCT. For example, an earlier clinical trial compared treatment responses to 3 treatment options for depression: antidepressant medication (nefazodone, *n* = 226), psychotherapy (non-MBI, *n* = 228) and combined nefazodone & psychotherapy (*n* = 227), with >30% of patients having experienced at least one type of childhood trauma in each arm, and found that patients with childhood trauma responded significantly better to psychotherapy than antidepressant medication (Nemeroff et al., 2003). There have not been RCTs of this size in the MBI literature with pre-determined ACE level stratification. One recent RCT reported MBI (*n* = 102) was *not inferior* to escitalopram (*n* = 106) for treatment of anxiety, but there was no ACE information in this RCT (Hoge et al., 2023). One prior 3-arm RCT compared MBCT (*N* = 99) vs. psychoeducation active control (*N* = 103) vs. treatment as usual (*N* = 53), and found MBCT overall did not have superiority over either control condition in preventing relapse of recurrent depression, but only among patients with above-median-level of childhood trauma, MBCT demonstrated superiority compared to the other two treatment options (Williams et al., 2014). Although we did not find significant difference between the two treatment arms among the subset of patients with above-median-levels of ACE, due to the small sample size and MBI program used in the current study not designed for treating chronic pain, we do not have the statistical power or clinical confidence to support this negative conclusion. To answer this question, we need a new clinical trial, with an MBI specifically focused on addressing self-criticism and pain-related psychological symptoms, with a well-powered sample size, and pre-designed stratification with ACE levels in the randomization procedure of the RCT.

Mechanistically, MBI vs. control appeared to have different psychological pathways. The MBI arm showed a direct path of SCR to pain catastrophizing, whereas in the control arm, SCR affected pain catastrophizing indirectly through depression. The objectives and contents of the two intervention programs were very different. The primary objectives of the MBI program were to enhance mindfulness to reduce craving and prevent relapse, whereas the objective of the control program was to provide peer support and convey evidence-based skills for OUD recovery. Teaching in MBI emphasized “*mindfulness*” concepts and practices (Rosansky et al., 2024), e.g., focusing on the present moment (instead of ruminating about the past or worrying about the future), cultivating body awareness (which also facilitates present moment awareness), and non-judgmental responses to external and internal stressors, all of which can alleviate common psychological symptoms among patients with high ACE such as rumination (Domke et al., 2023; Mansueto et al., 2021) and self-criticism (Glassman et al., 2007; Lassri and Gewirtz-Meydan, 2022), with the overlap of the two aspects being captured by the SCRS questionnaire used in this study (Smart et al., 2016). Especially, the “non-judgmental” responses style and self-compassionate responding cultivated in the MBI can be particularly helpful for reducing SCR (Neff, 2016). In contrast, the control group focused on skill development and peer support, which was shown in prior research to be very efficacious for reducing depression (Pfeiffer et al., 2011; Shorey and Chua, 2023). Prior research has demonstrated that peer support can improve depressive symptoms through reducing isolation, blunting the impact of stressors and sharing self-help knowledge (Dennis, 2003; Pfeiffer et al., 2011). The control arm in this study also showed significant symptom reduction of SCR, depression, pain catastrophizing and pain interference, likely because of the effects of peer-support on depression and development of coping skills.

Findings from the present study suggest SCR can be considered as a potential therapeutic target for reducing psychological and functional pain-related outcomes, such as pain catastrophizing and pain interference. Pain catastrophizing refers to the negative cognitive and affective response to anticipated or actual pain (Quartana et al., 2009), such as rumination, magnification and helplessness (Craner et al., 2016). Pain interference refers to the degree to which pain limits or interferes with individuals’ physical, mental and social activities (Amtmann et al., 2010); it is a holistic measure of the psychosocial impact of pain and is frequently used as an outcome variable in trials of pain treatments. Previous research has demonstrated the detrimental impact of self-criticism on chronic pain prognosis (Rudich et al., 2008) and treatment outcomes (Kempke et al., 2014). A recent RCT with cognitive behavioral therapy focused on rumination demonstrated reduced depression, anxiety and pain severity among patients with chronic lower back pain (Soleymani et al., 2020). Data from the present study suggests SCR can either be reduced directly via learning mindfulness especially by practicing a “non-judgmental” and “self-compassionate” response style to internal and external stressors, or indirectly through receiving peer group support and skills training to reduce the sense of isolation and co-morbid depression symptoms. With the advent of precision psychiatry (Fernandes et al., 2017; Passos et al., 2022), findings from this study may inspire development of personalized interventions, for customizing MBIs for individuals or sub-populations with certain psychosocial characteristics such as the types and severity of ACEs.

This study has several major limitations: (1) The MBI used in the present study (M-ROCC) was not specifically designed to target SCR nor pain interference, it was designed to support addiction recovery from OUD via cultivating mindful awareness of craving and prevent relapse (Rosansky et al., 2024), which appeared to have alleviated SCR through the general effects of improving psychological traits for mindfulness and self-compassion. Future studies shall consider adapting MBIs to specifically target SCR and pain interference. (2) The relatively homogenous demographic characteristics of the study sample imposed major limitations with the generalizability of the findings. This sample is representative of patients who had access to buprenorphine treatment for OUD, which does not represent other patients with OUD who do not have access to treatment (Beetham et al., 2019; Robbins et al., 2021), which has been a major health equity issue in public health (Lagisetty et al., 2019). The racial composition of the sample is mostly white, which limits the generalizability of our findings for other racial and ethnic populations (Paine et al., 2021). The intervention was developed and delivered in English, which may not directly generalize to other languages, for which cultural and linguistic adaptation may be required. (3) As a secondary data analysis study, this study was constrained by the study sample and variables collected in the parent RCT, which limited explorations of other potential mechanisms; the sample size is also underpowered for path analysis or other multivariate statistical modeling. Despite these limitations, the present study contributes to the literature in this research area by highlighting the importance of reducing SCR as a key mechanism contributing to pain-related symptom improvements among participants undergoing non-pharmacological treatment and the distinctive way that MBIs modulate SCR in its role as a mediating variable between ACE and pain-related psychological symptoms.

## Data Availability

The data analyzed in this study is subject to the following licenses/restrictions: this manuscript reports results from secondary analysis of a previously collected dataset, data availability is subject to restrictions with the original dataset. Requests to access these datasets should be directed to DJ, djoss@challiance.org.
